# Reliability of the GAIN-SS, CRAFTT and PESQ screening instruments for substance use among South African adolescents

**DOI:** 10.4102/sajpsychiatry.v22i1.932

**Published:** 2016-07-15

**Authors:** Tara Carney, Bronwyn Myers, Johann Louw

**Affiliations:** 1 Alcohol, Tobacco and Other Drug Use Research Unit, South African Medical Research Council, South Africa; 2 Department of Psychiatry and Mental Health, University of Cape Town, South Africa; 3 Department of Psychology, University of Cape Town, South Africa

## Abstract

**Introduction:**

Screening for adolescent substance use can assist with the early identification of substance-related problems and guide the provision of appropriate services. As such, psychometrically sound screening tools are needed. The aim of this study was to compare the reliability of the CRAFFT, Global Appraisal of Individual Needs-Short Screener (GAIN-SS) substance use subscale and Personal Experience Screening Questionnaire (PESQ) among adolescents from disadvantaged communities in Cape Town, South Africa.

**Methods:**

Adolescents aged 12–19 years (*n* = 231) completed the three screeners at two points in time.

**Results:**

Findings show that all three of the screeners had adequate internal consistency (Cronbach α ≥ 0.8). Test-retest reliability was similar for all three screeners, with intraclass correlation coefficient values slightly higher for the PESQ (0.82, 95% CI: 0.77–0.86) than for the GAIN-SS substance use subscale (0.79, 95% CI: 0.73–0.84) and CRAFFT (0.76; 95% CI: 0.66–0.83). Kappa values indicated that the GAIN-SS substance use subscale and CRAFFT had moderate levels of agreement, while the PESQ had substantial levels of agreement for identifying those who had moderate or higher substance use risks at Time 1 and Time 2.

**Conclusion:**

The findings indicate that all of these short screeners seem to have acceptable reliability when used in this population. All of the three screeners are appropriately reliable when used with adolescents from disadvantaged communities in Cape Town, but the PESQ performed slightly better. Future studies should also include the assessment of validity of these screeners in this context.

There is a high prevalence of substance use among adolescents in the Western Cape, with up to two-thirds of adolescents in this province reporting a lifetime use of at least one substance.^1,2^ Adolescents in this province report using a broad range of substances, with alcohol and tobacco being the most commonly reported, followed by cannabis and methamphetamine.^3^ The high prevalence of substance use among adolescents in this region is cause for concern as adolescence is a sensitive developmental period^4,5^ and early initiation of substance use could negatively impact on neurocognitive development.^6^ In addition, adolescent substance use has been associated with poor physical health,^7^ mental health,^8,9^ and academic outcomes^10^ as well as engagement in other risk behaviours,^2,9,11^ all of which may negatively impact on future well-being.^12^

Consequently, it is vital to identify adolescents who may be using substances so that those at risk for adverse consequences can be provided with preventative interventions that reduce the risk of harms associated with continued substance use involvement.^13^ There is accumulating evidence that brief interventions are effective options for adolescents who have mild-to-moderate substance use problems but may not yet require intensive treatment.^14,15,16,17^ However, to realise the promise of these brief interventions, adolescents who may benefit from these interventions first need to be detected. Universal screening of adolescents in healthcare settings has been recommended as a method for identifying adolescents with potential substance use problems.^18^

For universal screening of adolescents to be effective, self-report screening tools need to be reliable for this subpopulation.^13,14,15,16,17,18^ Using a reliable screener means that it should be relatively free from error and consistent in its measurement. Two types of reliability are usually measured for screening tools: internal consistency and test-retest reliability. A screener with acceptable levels of internal consistency implies that responses to the different items in the instrument are similar to each other,^19^ while for test-retest reliability, the results of the screener administered at two different times are the same unless there has been an actual change in their behaviour.^19,20^ If a screener is reliable over time, one could advise service providers that the tool is an accurate reflection of problem behaviours.

Screening tools should also be effective in detecting those at risk of and actively using substances,^18^ so that they can differentiate between adolescents who are and are not at risk for substance use at various time periods. In the United States, several screening tools developed to detect potential substance use problems among adolescents have been shown to be psychometrically sound. For example, the Global Appraisal of Individual Needs-Short Screener (GAIN-SS) is a brief self-report screening tool that was developed from a standardised clinical interview.^21^ US studies have shown that the GAIN-SS performs well in comparison with standardised clinical interviews, demonstrating acceptable internal consistency, and is able to accurately identify adolescents who use substances.^22^

The CRAFFT is another brief screening tool that has acceptable reliability and validity among US adolescent populations.^23,24,25,26^ It comprises six questions examining substance use and risk behaviours associated with substance use,^5^ which are simple to score and easy to remember.^6^ In US adolescent populations, the CRAFFT appears to have adequate levels of test-retest reliability and internal consistency,^27^ but internal consistency findings in other countries have been equivocal.^28,29^

Another screener that has been developed from a more comprehensive clinical assessment is the Personal Experience Screening Questionnaire (PESQ). Items in the PESQ derive from the Personal Experience Inventory.^13,30^ The PESQ is not as well-studied as other screeners. Two studies using diverse US adolescent populations have demonstrated that the PESQ has acceptable levels of reliability and validity.^31,32^

While all three of these screeners seem appropriate to use among US populations, the extent to which these screeners are helpful for identifying adolescents in developing country settings, such as South Africa, who may be at risk for substance use disorders remains unknown. For example, although the GAIN-SS and CRAFFT are increasingly utilised as screeners for substance use in South Africa,^31,32^ their psychometric properties have not been established for local populations, and it is unclear whether findings from US studies can be extrapolated to South Africa.^33,34^ South African adolescents have lower levels of literacy^35^ and educational attainment^36^ relative to their counterparts in developed countries. This may impact on how South African adolescents respond to the items contained in these screeners and consequently their sensitivity and specificity for this population. Before these screening tools can be recommended for use among South African adolescents, more research is needed to establish their psychometric properties for this population.

## Objectives

The aim of this study was to examine the relative performance of the GAIN-SS, CRAFFT and PESQ among a sample of South African adolescents. Specifically, we set out to establish the internal consistency reliability and test-retest reliability of these screeners.

### Method

This study used a repeated measures design with two data collection time points.

### Sample characteristics

Participants were recruited using convenience sampling. Adolescents were recruited from 15 community-based organisations providing programmes for at-risk adolescents from several economically disadvantaged communities in Cape Town. To be included in the study, participants had to be aged between 12 and 19 years, attending school (or the equivalent of school) and able to understand English. EpiCalc 2000 was utilised to calculate the sample size that would be needed to have least 80% power, allowing for a 5% error margin rate. Post-hoc power calculations were consequently done with the following results. In a test for agreement between two raters using the Kappa statistic, a sample size of 260 subjects achieves 90% power to detect a true Kappa value of 0.65 in a test of H0: Kappa = κ0 versus H1: Kappa ≠ κ0 when there are three categories with frequencies equal to 0.50, 0.30 and 0.20. This power calculation is based on a significance level of 0.05 (Table 3).

### Measures

#### The set of screeners included the GAIN-SS, CRAFFT and PESQ

GAIN-SS: In addition to demographic questions, the GAIN-SS comprises 20 questions grouped into four subsections, which address internalising disorders (mental health issues such as depression and anxiety), externalising disorders (mental health issues related to attention and behaviour), substance use disorders (abuse, dependence and problems resulting from any kind of substance use) and engagement in crime or violent behaviours (interpersonal violence and criminal behaviour). Each subscale consisted of five questions.^21,22^ The current study, however, only examined the substance use subscale in order to compare the psychometric properties of this subscale relative to the other screeners. Respondents are asked to rate the frequency of symptom/behaviour occurrence, with responses rated on a five-point Likert scale from ’never’ (score: 0), ’more than a year ago’ (score: 1), ’7–12 months ago’ (score: 2), ’1–6 months ago’ (score: 3) to ’in the past month’ (score: 4) to provide a clear idea of how recently behaviours occurred in this study. The recommended cut-off point for the substance use subscale is 1, which indicates moderate use and a need for brief intervention or outpatient intervention. A score of three or higher indicates a probable diagnosis but with a requirement for a formal assessment and intervention.^22^

CRAFFT: The CRAFFT includes questions about past year alcohol use, cannabis use and other drug use (Section A) and six questions on the consequences of substance use (Section B).^26^ Responses are ’yes’ or ’no’ for all items. Each ’yes’ answer in Section B is scored as 1, with a total score of two indicating that further assessment of substance use involvement is warranted.^25^

PESQ: A modified version of this screener was developed to include demographic questions on race, gender, current grade and age. The screener includes 18 items that ask about substances used and the consequences of substance use. Responses to these items range from ’never’ (score: 1), ’once or twice’ (score: 2), ’sometimes’ (score: 3) or ’often’ (score: 4). Cut-off scores of 23 and 24 are used to identify younger (aged 12–15 years) and older (aged 16–18 years) adolescents, respectively, who would benefit from a brief substance use intervention. However, younger adolescents who score at least 30, older adolescent females who score at least 34 and older adolescent males who score at least 35 should receive a comprehensive assessment as they may have a substance abuse or dependence problem.^30^

## Procedure

Ethics approval for this study was obtained from the Faculty of Health Sciences at the University of Cape Town. Community organisations were approached, and informed about the study. At those organisations that agreed to participate in the study sessions, appointments were arranged for administering the screeners to eligible participants at Time 1 (T1) and 2 days later, at Time 2 (T2). This short time period was selected to avoid measurement over the weekend, which is the most likely time that adolescents used substances. This ensured that the screeners were not measuring any actual behavioural differences. Before the screeners were administered, we obtained informed consent to participate in the study from parents and (separately) from the adolescents. After the informed consent process was completed, a project staff member read the screeners aloud to the adolescents, item by item. Participants then completed the screeners themselves in a private setting approximating examination conditions. Each participant was given a unique identifying number which was placed on the screener that they completed. This enabled questionnaires at T1 and T2 to be linked to a single participant. Screeners were provided in English, with an Afrikaans and isiXhosa project staff member present to translate if necessary. The participants were provided with small incentives for their participation in the study. The screeners were administered in the same order at T1 and T2.

### Data analysis

Data were entered into Excel spreadsheets and checked for inconsistencies. It was then imported into SPSS (Version 22). Frequencies and descriptive statistics were calculated at T1 and T2. The psychometric evaluation of the instruments included the following:

1. **Test-retest reliability:** Intraclass correlation coefficients (ICCs) were computed to compare the results from T1 and T2. ICC values of 0.40–0.59 are considered fair, 0.60–0.74 considered to be good and 0.75 or more considered as excellent.^37^ Kappa coefficients were then used to compare the performance of the overall scale to the dichotomised scoring of the scale (those who scored above and those who scored below the cut-off scores). Moderate agreement is considered to apply to kappa values of 0.41–0.6, substantial agreement to values between 0.61 and 0.80 while values over 0.81 are considered near perfect agreement.^37^
2. **Internal Consistency:** Cronbach’s alpha for the total list of items and for the subscales to measure internal consistency. Acceptable alpha values range from 0.70 to 0.95 for optimal levels of internal consistency.^38^

## Results

The sample size was 266 at T1, and 231 at T2, with 35 participants dropping out because of academic or sports commitments (*n* = 27) and a small number not providing a reason for drop out (*n* = 8). Participants were 56.3% female, 48.9% Black African and 48.5% ’mixed race’. The majority of participants were in Grade 9 (22.6%), followed by Grade 8 (18.7%). Over a quarter of the participants were still attending primary school (27.4%) and the mean age of participants was 15 (SD = 1.67; Table 1).

**TABLE 1:** Demographic characteristics of sample.

Variable	Demographic characteristics (*N* = 231) *N* (%)
**Gender**	
Female	130 (56.28)
Male	101 (43.72)
**Age**	
12	7 (3.03)
13	51 (22.08)
14	36 (15.58)
15	45 (19.48)
16	43 (18.61)
17	35 (15.15)
18	10 (4.33)
19	4 (1.73)
**Race**	
Black African	113 (48.92)
Mixed race	112 (48.48)
White people	3 (1.30)
Other	2 (0.87)
Indian	1 (0.43)
**Grade**	
5	5 (2.17)
6	22 (9.57)
7	36 (15.65)
8	43 (18.70)
9	52 (22.61)
10	23 (10.00)
11	30 (13.04)
12	19 (8.26)

*Source*: Authors’ own work

The mean score on the GAIN-SS substance use subscale was slightly lower at T1 (*M* = 2.73, SD = 3.65) than at T2 (*M* = 3.42, SD = 4.97). The difference was statistically significant (*t* = − 2.94, *df* = 230, *p* < 0.01). In terms of test–retest reliability and internal consistency, respectively, the ICC values (ICC: 0.79, 95% CI: 0.73–0.84) and the alpha values (α = 0.80*)* were high and very similar (Table 2). For the overall GAIN-SS scale (not shown in the table), the internal consistency at T1 was higher (α = 0.88*)* and the ICC was 0.87 (95% CI: 0.84–0.90). The mean score for the PESQ was slightly higher at T1 (*M* = 26.03, SD = 8.76) than at T2 (*M* = 24.43). This difference was statistically significant (*t* = 3.91, *df* = 230, *p* < 0.01). Again, the ICC (ICC: 0.82, 95% CI: 0.77–0.86) and alpha values (α = 0.83) were high. The mean score for the CRAFFT was also significantly higher at T1 (*M* = 1.96, SD = 1.55) than at T2 (*M* = 1.37, SD = 1.32; *t* = 6.27, *df* = 120, *p* < 0.01). The ICC value (ICC: 0.76, 95% CI: 0.66–0.83) and alpha values (α = 0.82) were similar.

**TABLE 2:** Reliability of the GAIN-SS, PESQ and CRAFFT.

Scale	Time 1: mean (SD, range)	Time 2: mean (SD, range)	ICC (95% CI)	Alpha
GAIN SS total substance use disorder	2.73 (3.65, 0–15)	3.42 (4.97, 0–20)	0.79 (0.73–0.84)	0.80
PESQ total	26.03 (8.76, 18–72)	24.43 (7.65, 18–53)	0.82 (0.77–0.86)	0.83
CRAFFT total	1.96 (1.55, 0–6)	1.37 (1.32)	0.76 (0.66–0.83)	0.82

*Source*: Authors’ own work

**TABLE 3:** Kappa levels of agreement above the cut-off score for substance use problems.

Screener	T1 *n* (%)	T2 *n* (%)	Kappa
GAIN-SS substance use	94 (40.7%)	94 (40.7%)	0.59*
CRAFFT	71 (30.7%)	57 (24.7%)	0.56*
PESQ	123 (53.2%)	95 (41.1%)	0.65*

*Source*: Authors’ own work

* The kappa value is significant.

The GAIN-SS identified 40.7% of participants with at least mild-to-moderate substance-related problems at both T1 and T2. The Kappa (*K*) values indicated that there was a moderate level of agreement between the two time periods (*K* = 0.59). For the CRAFTT, close to a third of the participants scored above the cut-off point for substance use problems at T1 (30.7%) but only a quarter scored above the cut-off point at T2 (24.7%). The Kappa (*K*) values indicated that there was a moderate level of agreement between the two time periods (*K* = 0.56). The PESQ also had a higher number of participants who scored above the cut-off at T1 (53.7%) in comparison with T2 (41.1%). The Kappa value was the highest of the three screeners (*K* = 0.65).

## Discussion

This study is the first to explore the psychometric properties of the CRAFFT, GAIN-SS and PESQ for use among South African adolescent populations. Findings from this study are potentially important, as these screeners are increasingly being used to detect adolescents who may benefit from substance use interventions,^35,36^ yet little is known about their reliability sc in adolescent populations from low- and middle-income countries. Findings from this study therefore provide preliminary insights into the cross-cultural applicability of these screeners.

Our findings suggest that while all three screeners have acceptable levels of temporal stability and internal consistency among South African populations. However, there were some differences among the screeners, and while the GAIN-SS and PESQ scored similarly, the CRAFFT seemed to have better internal consistency than test–retest reliability. The PESQ also seemed to have slightly higher test–retest reliability in the current study than the GAIN-SS and CRAFFT.

These findings are in agreement with findings from US studies which show that the GAIN-SS and PESQ have adequate psychometric properties. In terms of internal consistency, for example, findings from this study are consistent with studies examining psychometric properties of the CRAFFT,^23,26^ PESQ^30^ and GAIN-SS^22,38^ in terms of internal consistency in the United States. Findings from countries outside of the United States regarding the CRAFFT in comparison with other short screeners indicate that its reliability is lower than others,^28,29^ which was the case in the current study as well, albeit only very slightly. There only seems to be evidence on test–retest reliability on the CRAFFT,^27^ so this study is one of the first studies to look at temporal stability of a number of short screeners for adolescents.

In addition, even in this small sample of school-going adolescents in Cape Town, the average scores at both time points were above the cut-off scores for moderate risk of substance use problems. These findings are in line with previous South African studies that used the GAIN-SS^32^ and CRAFFT,^33^ although there have been no studies using the PESQ. This indicates the need for short screeners that show consistent performances with South African adolescents, in order to detect early problematic substance use and then provide timeous services. However, while the reliability values were acceptable, there were still differences between the proportions that scored above the cut-off points at the two time periods that were unaccounted for. Adolescents who score above the cut-off should first be referred for a comprehensive assessment that allows for potential substance use problems to be more thoroughly explored before being referred to intervention services after screening.

In summary, findings on the screeners’ performances suggest that GAIN-SS and CRAFFT are potentially suitable for use in this resource-poor context, because both have adequate psychometric properties, are brief, and are easy to administer and score.

**FIGURE 1:**
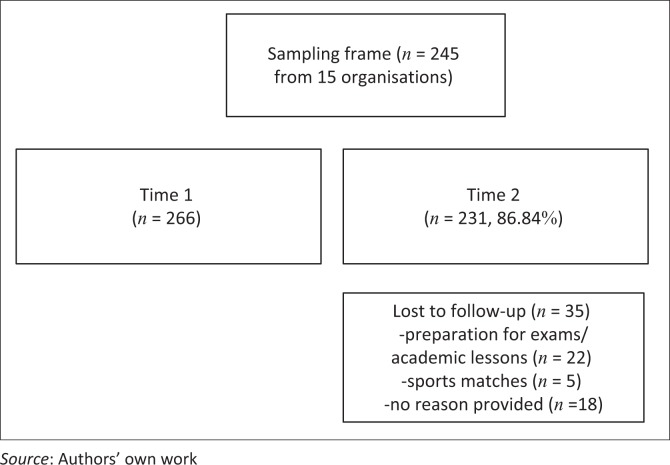
Flow diagram of screening process.

Nonetheless, these findings should be considered in the light of several methodological limitations. Firstly, it was not possible to compare the three screeners with a standardised diagnostic interview which is the gold standard for determining the diagnostic ability of short screeners. This is costly, and is beyond the resources of the current study. However, the GAIN-SS is based on the full GAIN interview and has acceptable psychometric properties that may be closest to a diagnostic interview, but this could not be tested in the current study.^40^ Secondly, all three of the screeners were originally developed in English. Despite the training provided to research staff and the thorough instructions provided to adolescents in their indigenous language, it is possible that some of the adolescents would have had a better comprehension of the screening questions if they were provided in their home language. Future studies should consider translating these screeners into the other two indigenous languages most commonly utilised in the Western Cape and assessing the psychometric properties of these language versions. Finally, it is possible that some of the adolescents simply recalled their responses given at Time 1 and again at Time 2, since it was only 2 days later. Further research that extends the interval between Time 1 and Time 2 should therefore be conducted, to compare if there are differences between the results with a longer time period (Figure 1).

## Conclusion

In conclusion, our findings suggest that the GAIN-SS, PESQ and CRAFFT are appropriate for use with South African adolescents, although the CRAFFT seemed slightly less reliable than the other two screeners. While it is important to measure reliability, future studies should also consider measuring these screeners against a standard diagnostic tool in order to measure validity, and obtain a comprehensive picture of the instruments’ psychometric properties. However, this is an important step in identifying appropriate tools for the early detection and intervention of substance-using adolescents.
